# Dynamic changes in platelets caused by shear stress in aortic valve stenosis

**DOI:** 10.3233/CH-200928

**Published:** 2021-02-12

**Authors:** Hideaki Kanda, Munekazu Yamakuchi, Kazuhisa Matsumoto, Kosuke Mukaihara, Yoshiya Shigehisa, Shuji Tachioka, Masashi Okawa, Kazunori Takenouchi, Yoko Oyama, Teruto Hashiguchi, Yutaka Imoto

**Affiliations:** a Cardiovascular and Gastroenterological Surgery, Kagoshima University Graduate School of Medical and Dental Sciences, Kagoshima, Japan; b Department of Laboratory and Vascular Medicine, Kagoshima University Graduate School of Medical and Dental Sciences, Kagoshima, Japan

**Keywords:** Aortic valve stenosis, platelet, plateletcrit, shear stress, pressure gradient

## Abstract

**BACKGROUND AND OBJECTIVE:**

Turbulent blood flow in patients with aortic valve stenosis (AS) results in morphological and functional changes in platelets and coagulation factors. The aim of this study is to determine how shear stress affects platelets and coagulation factors.

**METHODS:**

We retrospectively evaluated data from 78 patients who underwent AVR to treat AS between March 2008 and July 2017 at Kagoshima University Hospital.

**RESULTS:**

Platelet (PLT) count obviously decreased at three days after AVR, and increased above preoperative levels at the time of discharge. In contrast, platelet distribution width (PDW), mean platelet volume (MPV), and platelet large cell ratio (P-LCR) increased three days after AVR, then decreased to below preoperative levels. No differences were evident between groups with higher (HPPG > 100 mmHg) and lower (LPPG < 100 mmHg) peak pressure gradients (PPG) before AVR, whereas PLT count, PDW, MPV and P-LCR improved more in the HPPG group. Plateletcrit (PCT), which represents the total volume of platelets, increased after AVR due to decreased shear stress. High increasing rate of PCT was associated with lower PLT count, higher PDW and lower fibrinogen.

**CONCLUSION:**

Shear stress affects PLT count, PDW, and fibrinogen in patients with AS.

## Abbreviations

AVR: Aortic valve replacement
AS: Aortic valve stenosis
PPG: Peak pressure gradient(s)
HPPG: High peak pressure gradient(s)
LPPG: Low peak pressure gradient(s)
MPV: Mean platelet volume
PCT: Plateletcrit
PLT: Platelet(s)
PDW: Platelet distribution width
P-LCR: Platelet large cell ratio
TAVI: Trans-catheter aortic valve implantation

## 1 Introduction

The prevalence of cardiovascular diseases associated with age-related vascular atheroma, intimal thickening, and calcification is increasing globally. Among valvular diseases, the number of patients with aortic valve stenosis (AS) caused by calcification has also increased, and it is expected to double within the next 20 years. AS is generally treated by replacing a stenotic aortic valve with a prosthetic valve, which requires a cardiopulmonary bypass during the procedure and causes problems for older patients. Trans-catheter aortic valve implantation (TAVI) is less damaging for patients with AS. However, this is sometimes contraindicated due to anatomical restrictions and financial issues. At present, the pathological mechanisms of AS and valve calcification have not been sufficiently elucidated, and preventive agents are unknown. Valve calcification gradually progresses in AS, and the area of valve orifices decreases over time. As the pathological lesion advances and the valve orifice area decreases, blood flow passing through the aortic valve becomes turbulent [1], and peak pressure gradient (PPG) determined by echocardiography increases. Accelerated blood flow accompanying these events might influence hemodynamics.

Shear stress due to blood flow acts in a tangential direction to the surface of blood vessels and increases according to blood viscosity and flow velocity [2]. Shear stress is thought to affect the endothelium and vascular smooth muscle as well as blood cells, and change their function [3, 4]. Shear stress can increase nitric oxide release from endothelial cells and erythrocytes [5]. The permeability and anticoagulant ability of the vascular endothelium become altered, resulting in disorders associated with atherosclerosis, and caused vascular calcification [6]. Shear stress in AS also causes changes in von Willebrand factor (vWF) that result in Heyde syndrome, which is characterized by abnormal vessel neogenesis and bleeding in the intestinal tract [7]. Similarly, shear stress can alter platelet activity *in vivo* and *in vitro* [8]. For instance, shear stress increased plasma transforming growth factor-beta (TGF-*β*) by activating platelets in mice AS model [9]. Plateletcrit (PCT), representing the total platelet volume in the blood, has been shown to be associated with coronary artery diseases [10], however, the dynamics of PCT in AS patients have not been fully studied.

Aortic valve replacement (AVR) normalizes turbulent blood flow caused by AS, and should thus alter force and direction of shear stress. That is, shear stress before and after AVR might be quite different, and exert various effects on blood cells. This study analyzed changes in the peripheral blood before and after AVR and examined the influence of shear stress on blood cells, especially on platelets.

## 2 Methods

### 2.1 Patients and clinical data collection

We retrospectively investigated 78 patients who underwent AVR only at our hospital between March 2008 and July 2017. We collected blood test results, clinical information about the patients, and transthoracic echocardiography data. Peripheral blood data were collected before surgery (T1) and on postoperative days (POD) 3 (T2) and 7 (T3), and on the day of discharge (T4). Biochemical and coagulation data were collected at T1 and T4. The first postoperative month is described as T4 for patients who remained in hospital for more than one month. We compared white blood cell (WBC) count, red blood cell (RBC) count, RBC distribution width (RDW), platelet (PLT) count, platelet distribution width (PDW), mean platelet volume (MPV) and platelet large cell ratio (P-LCR) in peripheral blood samples. The patients were assessed by transthoracic echocardiography before surgery. We measured the PPG, mean pressure gradient (MPG), aortic valve area and left ventricular ejection fraction (LVEF). No case was excluded to use analyzed data in this study. This investigation proceeded under approval from the Kagoshima University Hospital Clinical Research Ethics Committee (approval number; 180227). The study was conducted in accordance with the ethical standards of the Committee on Human Experimentation of the institution at which the experiments were performed or in accordance with the ethical standards of the Helsinki Declaration of 1975.

### 2.2 Statistical analysis

Data were statistically analyzed using GraphPad Prism 8 (GraphPad Inc, 2018). Values were expressed as means±SD. Groups were compared using *χ*^2^ tests, Student *t* tests or Mann-Whitney U tests, as appropriate. Values with *p* < 0.05 were considered to be statistically significant.

## 3 Results

We assessed data from 78 patients with AS, including 13 who were on hemodialysis. Table 1 summarizes their demographic and clinical characteristics. The preoperative PPG was 90.3±29.0 mmHg. The average postoperative stay in hospital was 23.9±16.5 days, and CRP (C-reactive protein) at discharge was 2.08±1.87 mg/dL. Fifty-six (71.8%), 26 (33.3%) and 47 (60.3%) patients had hypertension, diabetes mellites, and dyslipidemia, respectively.

**Table 1:** Patient characteristics

All patients	78
Male: female	43: 35
Age	71.9±9.1
Body mass index (BMI)	23.1±4.27
Body surface area (BSA) (m^2^)	1.53±0.18
Casual factors
Hypertension	56 (71.2)
Diabetes mellitus	26 (33.3)
Dyslipidemia	47 (60.3)
Smoking	30 (38.5)
Hemodialysis	13 (17.7)
Echocardiographic parameters
Left ventricular ejection fraction (LVEF) (%)	61.4±13.9
Peak pressure gradient (PPG) (mmHg)	90.3±29.0
Aortic valve area (cm^2^)	0.66±0.19
Laboratory data
T-Bil (mg/dL)	0.76±0.39
AST (U/L)	23.5±7.89
ALT (U/L)	19.4±14.2
Cre (mg/dL)	0.89±0.38
CRP (mg/dL) at discharge	2.08±1.87

Values are mean±SD, or n (in percent).

**Fig. 1:**
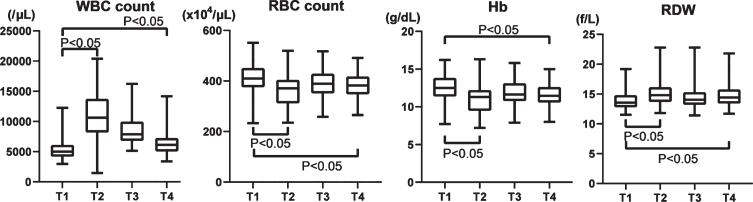
Changes of WBC, RBC, Hb, and RDW in the perioperative period of aortic valve replacement. WBC: White blood cell, RBC: Red blood cell, Hb: hemoglobin, RDW: Red blood cell distribution width.

WBC count increased more than twice from 5,505±1,920/*μ*L at T1 to 10,772±3,847/*μ*L on T2 and gradually decreased to 6,534±2,242/*μ*L at T4, but not to the preoperative level (Fig. 1). RBC counts and hemoglobin (Hb) temporarily decreased on T2 and then gradually recovered, but not quite to preoperative levels (T1, 410±60.8×10^4^/*μ*L; T2, 366±64.4×10^4^/*μ*L; T3, 389±55.4×10^4^/*μ*L; and T4, 381±50.7×10^4^/*μ*L; Fig. 1). The RDW inversely varied, increasing at T2 and partially recovering by T3 and T4 (T1, 14.0±1.67 f/L; T2, 15.1±1.89 f/L; T3, 14.5±1.80 f/L; T4, 14.8±1.89 f/L; Fig. 1).

**Fig. 2:**
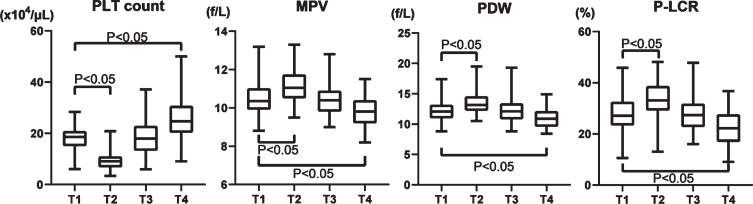
Changes of four platelet factors in the perioperative period of aortic valve replacement. PLT: platelet, MPV: mean platelet volume, PDW: platelet distribution width, and P-LCR: platelet large cell ratio.

Platelet count decreased by about 50% postoperatively (T1, 18.3±4.69×10^4^/*μ*L and T2, 9.64±3.56×10^4^/*μ*L), increased to 18.0±6.65×10^4^/*μ*L at T3, and reached 25.5±8.21×10^4^/*μ*L at T4, which exceeded that at T1 (Fig. 2). MPV increased on T2, then decreased on T3 and T4 (T1, 10.5±0.91 fL; T2, 11.2±0.86 fL; T3, 10.4±0.86 fL; and T4, 10.5±0.91 fL) (Fig. 2). Notably, the PDW increased on T2 (T1, 12.2±1.63%; and T2, 13.6±1.88%), but it decreased below the preoperative level at one month after the procedure. (T1 vs. T4, 12.2±1.63% vs. 10.9±1.40%; Fig. 2). P-LCR also increased on T2, but decreased at T4 (T1, 27.6±7.33%; T2, 33.8±6.98%; T3, 28.0±7.18%; T4, 22.4±6.44%) (Fig. 2).

**Fig. 3:**
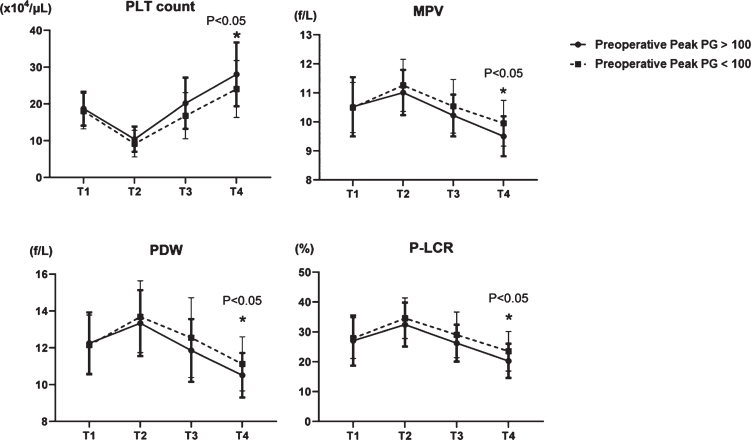
Comparison of four platelet factors in two groups; HPPG (Peak PG >100 mmHg) vs LPPG (Peak PG <100 mmHg). PLT: platelet, MPV: mean platelet volume, PDW: platelet distribution width, and P-LCR: platelet large cell ratio.

We analyzed relationships between platelets and pressure gradients using echocardiography. The pressure gradient (PG) can be calculated from flow velocity according to the simple Bernoulli equation (*Δ*P (mmHg) = 4× (velocity (m/s))^2^) and we assigned the patients into groups with HPPG (>100 mmHg, *n* = 28) and LPPG (<100 mmHg, *n* = 50). Four platelet factors (PLT count, MPV, PDW, and P-LCR) were not changed at T1 in the two groups (Fig. 3). Although decreased at T2 in both groups, PLT count increased more in the group with HPPG, than with LPPG at T4 (Fig. 3). In contrast, MPV, PDW, and P-LCR were increased at T2 in both groups and diminished more at T4 in the group with HPPG (Fig. 3). In PG classification, there was no difference in preoperative values, but there was a difference in postoperative platelet morphology, probably due to differences in shear stress.

**Fig. 4:**
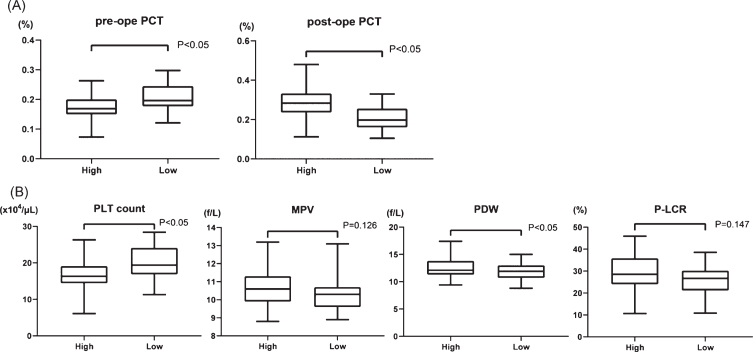
(A) Comparison of PCT (plateletcrit) values in two groups; high PCT increasing rate (high) vs low PCT increasing rate (low) before and after surgery. (B) Comparison of four platelet factors between high and low group before surgery. PLT: platelet, MPV: mean platelet volume, PDW: platelet distribution width, and P-LCR: platelet large cell ratio.

To establish a new factor with which to predict the extent of shear stress, we calculated plateletcrit (PCT (%); PLT count (10^4^/*μ*L)×MPV (fL)×10^–3^), which represents the total platelet volume in the blood, before and after surgery. The transition of PCT had no significant difference between HPPG and LPPG (Supplementary Figure 1). Therefore, we assigned the patients in two groups according to whether they had a high rate of increase PCT (high PCT, 39 patients) or a low rate of increase PCT (low PCT, 39 patients). The preoperative and postoperative PCT increasing rates were 0.175±0.041% and 0.282±0.073%, respectively, in the group with high PCT and 0.207±0.048% and 0.211±0.059% in the group with low PCT, respectively (Fig. 4A). We then compared preoperative platelet factors, PLT count, MPV, PDW and P-LCR, between the groups according to the increasing rate of PCT (Fig. 4B). The PLT count was 16.5±4.09×10^3^/*μ*L and 20.0±4.58×10^3^/*μ*L (*p* < 0.001) in the groups with high and low PCT increasing rate, respectively. In contrast, PDW was 12.6±1.74% and 11.8±1.41% (*p* = 0.04) in the groups with high and low PCT increasing rate, respectively. Neither MPV nor P-LCR significantly changed (10.7±0.936 fL vs. 10.3±0.853 fL, *p* = 0.13; 28.8±7.98% vs. 26.4±6.39%, *p* = 0.15, respectively). These data suggest that high PCT, which should indicate high shear stress, results in smaller PLT count and larger PDW before AVR. In other words, the influence of shear stress to platelets was more powerful in patients with low PLT count and a large PDW before surgery.

**Fig. 5:**
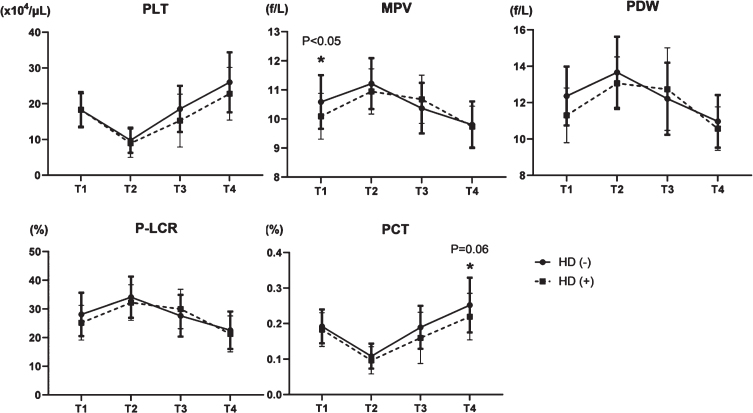
Transition of five platelet factors in patients not on hemodialysis (HD (–)) or dialysis patients (HD (+)). PLT: platelet count, MPV: mean platelet volume, PDW: platelet distribution width, P-LCR: platelet large cell ratio, and PCT: plateletcrit.

We compared platelet factors between patients on hemodialysis and not on hemodialysis to determine the impact of dialysis on the characteristics of platelets. The number of platelets transiently decreased in T2 and increased by T4 in both groups, and neither PDW nor P-LCR between both groups significantly changed after the procedure (Fig. 5). Although MPV was higher in the group not on hemodialysis in T1, this difference disappeared between T2 and T4 (Fig. 5). The PCT decreased in T2 and increased in T3 and T4, with slightly, but not significant (*p* = 0.06), different changes between the groups (Fig. 5). These data suggested that late recovery of PCT in dialysis group indicates more platelet damage, due to the effects of powerful shear stress from dialysis devices.

**Fig. 6:**
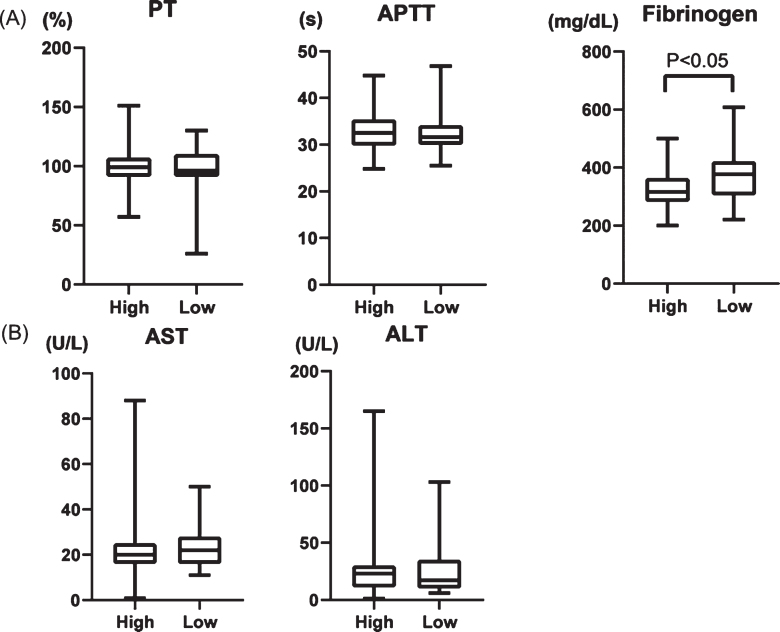
(A) Comparison of prothrombin time (PT), activated partial thromboplastin time (APTT), fibrinogen, (B) AST, and ALT in two groups; high PCT increasing rate (High) and low PCT increasing rate (low).

We evaluated laboratory findings of AST, ALT, fibrinogen, prothrombin time (PT), and activated partial thromboplastin time (APTT). Whereas PT and APTT did not significantly differ between patients with high and low PCT, the fibrinogen value was lower in the group with high PCT (Fig. 6A). The AST and ALT values did not significantly change in either group (Fig. 6B), suggesting that liver dysfunction does not cause lower fibrinogen levels.

## 4 Discussion

**Fig. 7:**
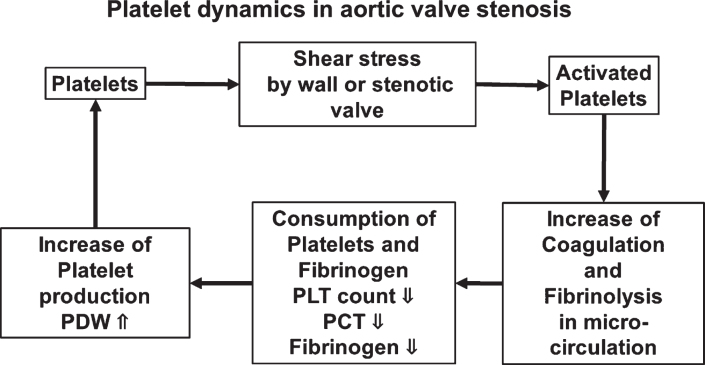
Schematic summary.

We assessed the dynamics of blood cells in patients with AS treated by AVR during the perioperative period. PLT count recovered after surgery, and PDW, MPV, and P-LCR, which represent variations in platelets, decreased compared with those before surgery. The preoperative PLT count was low and PDW was wide in the group with high PCT from which we predicted high shear stress. Among the patients with AS, PCT was a little lower for those on dialysis after AVR. Furthermore, fibrinogen was significantly low in the group with high PCT, suggesting a relationship between shear stress and coagulation ability in patients with AS (Fig. 7).

The frequency of bleeding increases after cardiac surgery due to the complexity and length of surgery and the need for a cardiopulmonary bypass. Prolonged postoperative bleeding requires more surgery, which negatively impacts mortality and increases the likelihood of further complications [11]. Vuylsteke et al. using Papworth Bleeding Risk scores, determined that undergoing surgery for aortic valve disease is a risk factor for bleeding [12]. Mazur et al. also showed that preoperative blood clots with highly permeable fibrin mesh was associated with a large volume of postoperative hemorrhage in patients with AS [13], suggesting that their coagulation ability is affected by hemodynamic stress. Heyde syndrome, namely intestinal bleeding due to abnormal angiogenesis in the digestive tract, often arises in patients with AS. The postulated molecular mechanism through which AS causes Heyde syndrome is that shear stress deforms spherical molecules of vWF, which is involved in platelet adhesion, from spherical to a linear form [14]. Interactions among vWF, platelet activities and coagulation activity might be involved in the onset of Heyde syndrome.

The intima of the blood vessels is constantly exposed to hemodynamic forces, namely vertical pressure from the circulating blood and fluid shear stress caused by tangential force and blood viscosity on the surface. Shear stress within the physiological range is required to maintain vascular function, but excessive shear stress damages blood vessels and blood cells [3], and is associated with cardiovascular pathologies such as myocardial infarction [15]. Accelerated, turbulent blood flow passing through a stenotic valve in AS causes excessive shear stress on the aorta wall and the aortic valve [1]. A means of using cardiac MRI to visualize and quantify shear stress is underway [16] and pressure gradients determined by echocardiography can be associated with shear stress by multiplication with flow velocity [7], however, these have not yet become a practical clinical application.

Shear stress activates leukocytes, leading to chronic inflammation in patients with AS [17]. We showed that WBC count significantly increased at T2 due to surgical invasiveness and recovered at T4, but not to the preoperative level. Since WBC count did not completely return to the preoperative value, a low level of inflammation might persist. We believe that the WBC count will decrease more over time. RBC count similarly decreased on T2 and returned to the preoperative level at T4. Procedural blood loss and blood transfusion volumes affect RBC counts, which interfered with the ability to determine the effects of shear stress on RBC in the present study [1]. Kawase et al. reported that AS causes hemolytic anemia [18], suggesting that RBC count will increase postoperatively. Neither WBC nor RBC change during one month of follow-up, therefore, further long-term follow-up is necessary.

On the other hand, the PLT count was higher at the time of discharge than before the operation, and other factors were lower than the preoperative value, and the change in platelets was larger than that of other blood cells. Regarding the blood of patients with AS, it was reported that the platelet and vWF activity was reduced before the operation and improved after the operation [19]. It was speculated that this platelet change was caused by AVR, which reduces shear stress. In addition, we considered that shear stress was related to the preoperative PPG on echocardiography and divided patients into groups with high and low PPG. Since severity of AS does not necessarily reflect the level of PG, postoperative values of platelet factors significantly differ although there's no differences in the preoperative values. Therefore, we analyzed PCT, which is the total volume of platelets in blood determined as MPV×PLT count [20]. We postulated that PLT would be consumed due to shear stress and PCT would be decreased before AVR, and that PCT would increase after surgery due to reduced shear stress. That is, we considered that the release from shear stress caused by AS had a more powerful effect in the group with a large PCT increase after surgery. In this group, the preoperative platelet count was low and PDW was broad, suggesting that shear stress might activate platelets, which would be consumed by creating micro-thrombus in peripheral circulation and newly-born platelets might be produced due to the decreased of platelet count.

Fibrinogen was low in high rate of increase PCT group, indicating that shear stress might influence coagulation ability. Natorska et al. have found high values for thrombin and PLT activities in some patients with AS [21], and suggested that coagulation and fibrinolysis caused by activated platelets in the microcirculation might deplete fibrinogen to exhaustion. They also argued that patients with AS had elevated plasma D-dimer and prothrombin fragment 1 + 2, and increased tissue factor in valve leaflets [22], which might also be associated with the coagulation system and progressive valve stenosis.

Shear stress between 100 and 1,000 dyn/cm^2^ activates circulating platelets. Since the estimated shear stress is 1,000–1,700 dyn/cm^2^ in AS mimic model [1], PLT will be easily activated in patients with AS. Activated platelets increased the secretion of TGF-*β* that promoted cell transformation in aortic valves [9] and enhanced blood vessel calcification in mouse models of atherosclerosis [23]. Shear stress also increased the release of extracellular vesicles (EV) or microparticles from platelets and leukocytes and the production of shear-destroyed platelets, leading to vascular inflammation [24, 25]. In turn, EV derived from vascular endothelial cells are constantly released by shear stress in patients with AS and this release is abrogated after TAVI, with subsequently improved vascular contractility [26]. Taken together, these findings indicate that the impact of shear stress on PLT contributes to the pathogenesis of AS.

AVR for AS requires a prosthetic valve, and mechanical or bioprosthetic valves are presently available, and the mechanical type requires the lifelong medication with warfarin, which causes vascular calcification [27]. Although reduced by AVR, shear stress is reportedly higher with prosthetic, than normal aortic valves [28]. In prosthetic valves deteriorate, one of the causes might be stress arising from the bloodstream, but another could be blood cells, including PLT, that become activated due to residual shear stress. Furthermore, the material of the implanted valve might influence the activation of the platelets in association with shear stress [8]. A future study of changes in blood cells associated with shear stress is important to improve the durability of bioprosthetic valves.

## 5 Limitation

This retrospective study is limited by the protocol design and a small patient cohort. Although restricted to patients who underwent AVR only, preoperative factors that could affect blood cells was impossible to standardize from their backgrounds. Moreover, since the patients were not followed up at our institution, changes beyond one month of follow-up could not be compared. We consider that high postoperative WBC counts and CRP values are due to a persistent inflammatory response to invasive surgery. Since inflammation might have affected blood cell counts and morphology, accurate longer-term comparisons of the effects of shear stress are warranted.

## 6 Conclusion

The present findings indicated that shear stress activated PLT, which were consumed by forming micro-thrombus and PCT might be a marker of shear stress in AS patients. The relationship between PCT and platelet function should be investigated in the future study.

## Conflict of interest

The authors declared no potential conflicts of interest with respect to the research, authorship, and/or publication of this article.

## Funding

The author(s) disclosed receipt of the following financial support for the research, authorship, and/or publication of this article: This research was supported by the following; Challenging Research (Exploratory) (18K19523) (M.Y.), Grant-in-Aid for Scientific Research (18H02734) (M.Y.), (16H05229) (T.H.), (19K09274) (Y.I.), (17K09310) (Y.O.), Grant-in Aid for Early-career scientists (18K16399) (H.K.), and (18K15424) (K.T.).
